# Lateral Column Realignment Combined With Anterior Longitudinal Ligament Release Versus Three‐Column Osteotomy in the Treatment of Thoracolumbar Kyphosis in Septuagenarians: A Retrospective Comparative Cohort Study

**DOI:** 10.1111/os.70176

**Published:** 2025-09-18

**Authors:** Xue‐Peng Wei, Hung‐Lun Hsieh, Qing‐De Wang, Yi‐Hsun Huang, Erh‐Ti Ernest Lin, Chen‐Wei Yeh, Yuan‐Shun Lo

**Affiliations:** ^1^ Department of Spinal Surgery Zheng Zhou Orthopaedic Hospital Zheng Zhou Henan Province China; ^2^ Department of Orthopaedic Surgery China Medical University Beigang Hospital, China Medical University Yunlin Taiwan China; ^3^ Department of Orthopaedic Surgery China Medical University Hospital, China Medical University Taichung Taiwan China; ^4^ School of Medicine, College of Medicine China Medical University Taichung Taiwan China

**Keywords:** adult thoracolumbar kyphosis, anterior longitudinal ligament (ALL), lateral column realignment (LCR), minimally invasive spine surgery, navigation‐assisted spine surgery, osteoporotic vertebral fracture (OVF), Oswestry disability index (ODI), sagittal imbalance, spinal fusion, three‐column osteotomy (3CO)

## Abstract

**Objective:**

Adult thoracolumbar kyphosis secondary to osteoporotic vertebral fractures (OVF) impairs the quality of life. Traditional 3CO provides correction but carries a high risk of complications, especially in the elderly. Minimally invasive anterior approaches may be safer. This study aims to compare the radiographic and clinical outcomes of septuagenarians with thoracolumbar kyphosis treated with single‐position navigated lateral column realignment with anterior longitudinal ligament release (LCR‐A) plus posterior column osteotomy (PCO) and posterior spinal fusion (PSF), or percutaneous pedicle screws (PPS) versus three‐column osteotomy (3CO).

**Materials and Methods:**

This retrospective study included 21 patients with LCR‐A and 54 with 3CO prospectively treated between March 2020 and April 2024. Radiographic parameters, the Oswestry Disability Index (ODI), SRS‐22 scores, complications, and perioperative data were analyzed over a 2‐year follow‐up period.

**Results:**

Although LCR‐A patients were older, they had significantly reduced blood loss, shorter operative times, and fewer fused levels than 3CO patients. LCR‐A achieved comparable deformity correction, with fewer complications, lower postoperative ODI, and better SRS‐22 scores. The LCR‐A group maintained radiographic correction, with fewer new neurological deficits and lower rates of infection, ileus, and delirium.

**Conclusions:**

Single‐position navigated LCR‐A is a safer and less invasive alternative to 3CO in elderly patients with thoracolumbar kyphosis, offering effective deformity correction, fewer complications, improved functional outcomes, and enhanced recovery.

**Level of Evidence:**

IV.

## Graphical abstract

Navigation‐assisted LCR‐A plus PCO/PSF offers effective correction of adult thoracolumbar kyphosis secondary to OVF. Compared with 3CO, LCR‐A demonstrated lower blood loss, shorter operative time, and fewer complications. Thus, LCR‐A may serve as a safer and less invasive alternative for elderly patients.

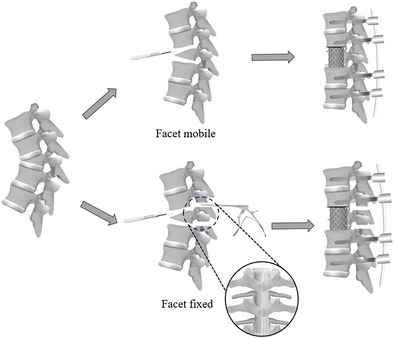

## 1 Introduction

Sagittal spino‐pelvic alignment, including pelvic position, is crucial for maintaining health‐related quality of life (HRQOL) in patients with adult spinal deformities (ASD) [1, 2, 3, 4]. Spinal sagittal malalignment correlates with pain, disability, and mental distress. With increased life expectancy, adult thoracolumbar kyphosis (ATLK) with sagittal imbalance, often due to osteoporotic vertebral fractures (OVF), has become more common. Traditional correction with three‐column osteotomy (3CO) carries high complication risks [5, 6, 7], especially in septuagenarians [8, 9, 10]. Recent studies have proposed that osteotomy via the anterior approach may offer distinct biomechanical and clinical advantages, such as preservation of anterior support, better control of the correction fulcrum, and reduced intraoperative bleeding [11, 12, 13]. Despite these theoretical benefits, anterior osteotomy plus realignment techniques have not been fully integrated into standardized osteotomy classification systems such as the SRS–Schwab classification, making their application and comparison less consistent across centers [14]. Furthermore, although various anterior osteotomy plus realignment techniques have been introduced in this decade—including anterior column realignment and lateral column realignment—the lack of comparative studies with PSO limits the establishment of evidence‐based indications [11, 13, 15, 16].

Therefore, there remains a critical gap in the literature regarding whether lateral column realignment with anterior longitudinal ligament release (LCR‐A) could provide comparable or superior clinical and radiographic outcomes to 3CO. Furthermore, recent advances in minimally invasive anterior spinal techniques and navigation‐assisted surgery have further reduced the invasiveness of anterior release procedures, while also eliminating the need for intraoperative repositioning, thereby saving operative time.

ATLK secondary to OVF is common in the elderly, with a prevalence of 9%–34% [17, 18], significantly impairing the quality of life. Although 3CO can effectively correct deformities, it has high complication rates. Okuda et al. [8] reported a 79% incidence of new vertebral fractures and 21% reoperation in 3CO patients (mean age: 73 years). Quarto et al. [9] found a 41.6% major complication rate, including pseudoarthrosis (20.9%), proximal junctional kyphosis (12.7%), dural tears (6.6%), and neurological deficits (3%). Bianco et al. [10] further noted significantly higher complication rates in patients aged > 60 years. With the advancement of minimally invasive anterior approaches that reduce the complications of conventional open procedures and the integration of navigated surgical systems that eliminate the need for intraoperative repositioning, the use of navigated LCR‐A emerges as a viable alternative to 3CO in the management of rigid kyphosis in the elderly population [11, 12, 13, 15, 16]. This study explored navigation‐assisted single‐position LCR‐A combined with posterior column osteotomy (PCO) and posterior spinal fusion (PSF) or percutaneous pedicle screws (PPS). We aimed to compare the radiographic and clinical outcomes of septuagenarians with thoracolumbar kyphosis treated with single‐position navigated LCR‐A plus PCO/PSF or PPS versus 3CO and to evaluate the efficacy and safety of the LCR‐A technique.

## 2 Materials and Methods

### 2.1 Subjects

This study was approved by the Institutional Review Board (CMUH111‐REC1‐128). Patients undergoing corrective spinal fusion with single‐position navigated LCR‐A plus PCO/PSF or PPS or 3CO for ATLK between March 2020 and April 2024 were included. ATLK was defined as kyphosis after OVF with C7 sagittal vertical axis (C7 SVA) > 50 mm, pelvic tilt (PT) > 25°, and/or thoracic kyphosis (TK) > 60°. The inclusion criteria were age > 65 years, complete preoperative full‐length radiographs, stable OVF under pillow test, and sagittal imbalance impairing standing. Exclusion criteria were neuromuscular disorders, spinal infections/tumors, and incomplete 2‐year follow‐up data. A total of 21 patients with LCR‐A and 54 patients with 3CO were analyzed.

### 2.2 3CO Procedure

We selected patients with kyphotic apex spinal cord shape types A and B to undergo 3CO procedures [19]. The 3CO procedures included pedicle subtraction osteotomy (PSO) and Schwab Grade IV osteotomy. The patients were positioned prone for abdominal decompression and table manipulation. Instrumentation extended at least three levels above and below the osteotomy site. For PSO, the spinous process and lamina of the targeted vertebra were removed. Adjacent segmental decompression was performed by excising the superior and inferior laminae. Under continuous irrigation with normal saline, a high‐speed burr was used to perform a transpedicular wedge osteotomy. Bone removal was carried out through both pedicles while preserving the medial walls as much as possible. The lateral and anterior cortices of the vertebral body were thinned using the burr, and a controlled wedge‐shaped cavity was created by resecting the posterior vertebral wall and a portion of the vertebral body. For Schwab Grade IV techniques, adjacent laminectomy and facetectomy at the level of osteotomy, and discectomy were performed. Subsequently, the base of transverse processes, the medial border, and the upper 2/3 of pedicles were resected, followed by resection of the posterosuperior corner of the target vertebral body. Wide disc space preparation for laterally was of utmost importance as it provided a broad field of view to facilitate bony resection and promote extensive bone‐to‐bone contact [20, 21, 22].

### 2.3 Lateral Column Realignment With ALL Release (LCR‐A) Procedure

**FIGURE 1:**
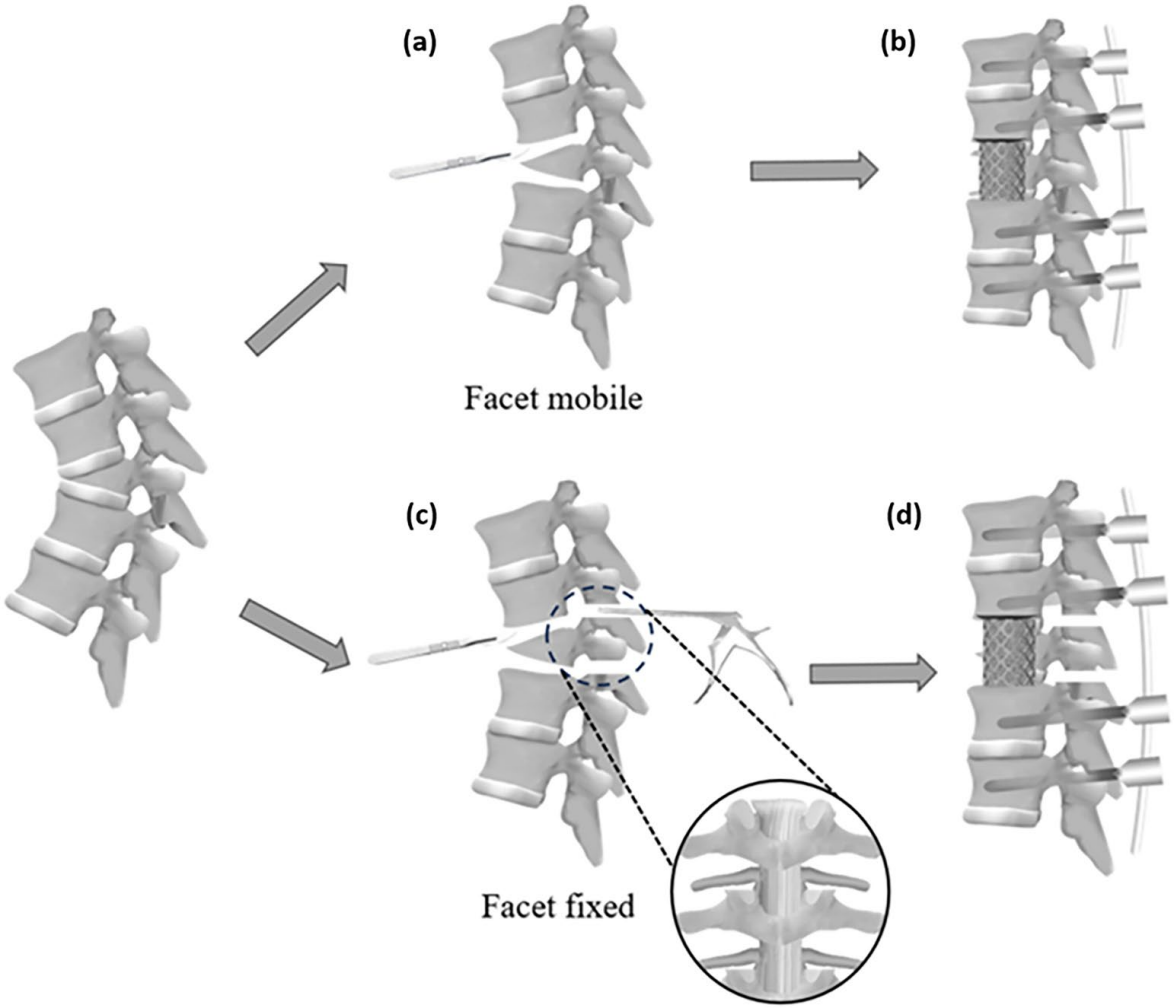
Schema describing single position navigated LCR‐A for thoracolumbar rigid kyphosis due to OVF (a) after ALL release and anterior osteotomy, facet mobile, (b) followed by PPS fixation under navigation; (c) after ALL release and anterior osteotomy, facet fixed, (d) followed by two level PCO–resection of the spinous process, bilateral inferior articular processes, and the caudal half of the lamina, as well as portions of the superior articular processes of the subjacent vertebra—and PSF under navigation. ALL: anterior longitudinal ligament; LCR‐A: lateral column realignment with ALL release; OVF: osteoporotic vertebral fracture; PCO: posterior column osteotomy; PPS: percutaneously pedicle screw; PSF: posterior spinal fusion.

Patients with kyphotic apex spinal cord shape types C− and C+ were selected to undergo the LCR‐A procedure [19]. The LCR‐A procedure was performed with the patients in the left lateral decubitus position using a sequential anterior–posterior approach. Under fluoroscopy, the anterior osteotomy segment was localized [23, 24]. A 3–4 cm rib segment was resected for autograft use, and retropleural dissection exposed the vertebral body while minimizing pleural injury. If below T12/L1, a minimal muscle‐splitting diaphragmatic incision was made below [23, 24]. After ligating the segmental artery, blunt dissection isolated major vessels from the anterior longitudinal ligament (ALL), with radiolucent retractors protecting the surrounding structures. Precise osteotomy planning and ALL release were performed using O‐arm navigation. Following disc removal and OVF resection, manual corrective pressure was used to assess the deformity mobility by applying to back gibbus. If sufficient, a mesh cage with autografts was implanted and PPS were placed in the lateral position under navigation. If mobility was inadequate, additional PCOs were performed before the final fixation. The PCO was performed across two segments, including the index level of the anterior osteotomy as well as the adjacent cranial level. The resection involved the spinous process, bilateral inferior articular processes, and the caudal half of the lamina at each level, as well as portions of the superior articular processes of the subjacent vertebra. Instrumentation was applied at a minimum of two levels above and two levels below the osteotomy site. The detailed procedure is shown in Figure 1.

The SYNMESH implant (DePuy Synthes), made of pure titanium and featuring an oblong shape with 5° press‐fit end rings, is specifically designed for the reconstruction of anterior column defects following anterior osteotomy at the thoracolumbar junction. Additionally, the minimally invasive pedicle screw system (Wiltrom Co. Ltd.), manufactured from titanium alloy (Ti–6Al–4V), incorporates a dual‐thread design and a cannulated structure to facilitate precise, guided insertion.

Neurophysiological monitoring with somatosensory evoked potentials (SSEP) and transcranial motor‐evoked potentials (Tc‐MEPs) was used in all cases. The perioperative surgical data collected included estimated blood loss (EBL), operative time (OPT), surgical procedure, number of instrumented vertebrae, and osteotomy level.

### 2.4 Radiographic Parameters

**FIGURE 2:**
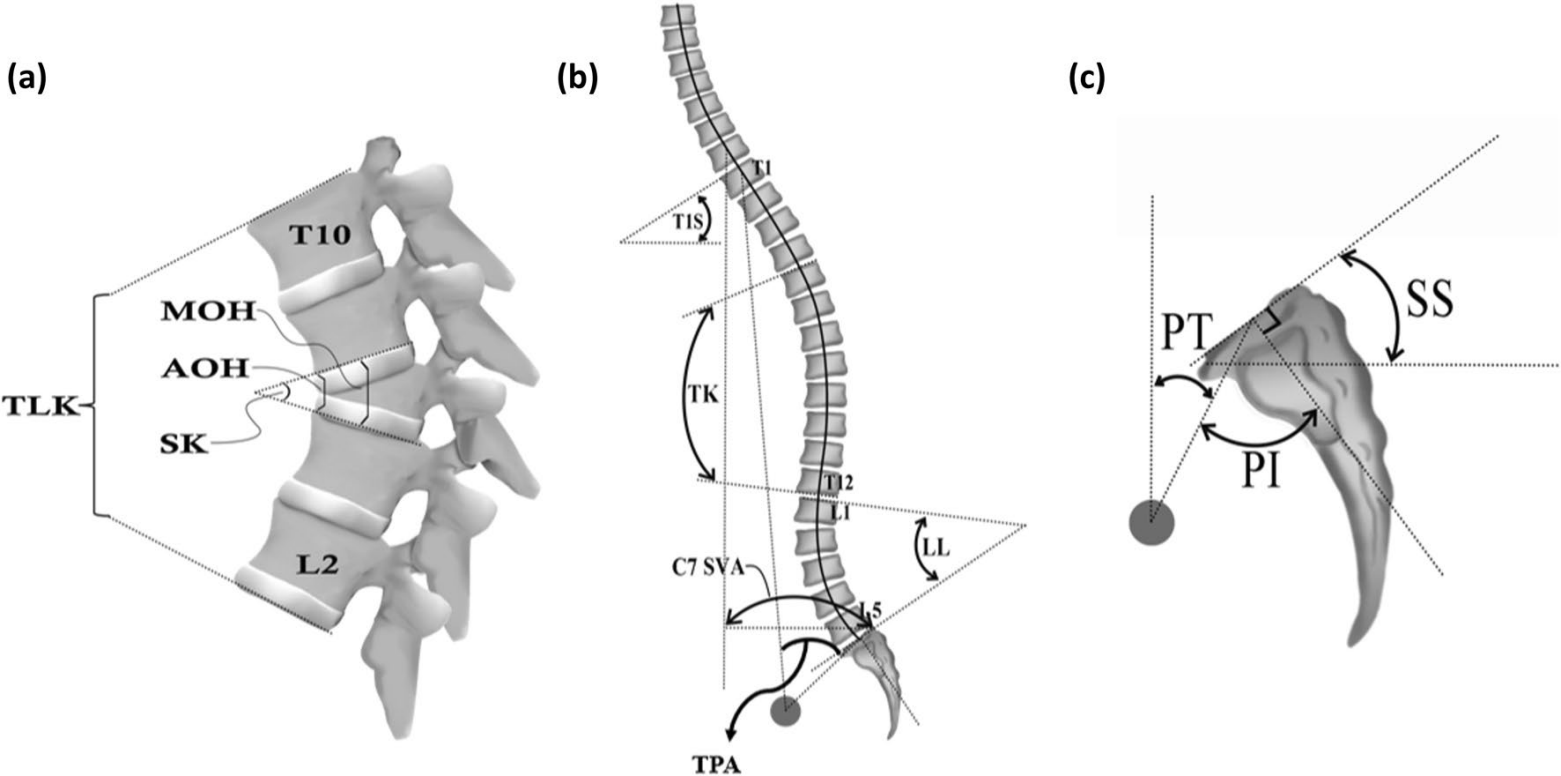
Illustration of measurements of spinal and pelvic parameters in sagittal radiograph, including (a) TLK, SK, AOH, and MOH (OH = (AOH + MOH)/2); (b) T1S, TK, LL, TPA, and C7 SVA; (c) PI, PT, and SS. (TLK: thoracolumbar kyphosis; SK: segmental kyphosis; AOH: anterior body height of osteotomy level; MOH: middle body height of osteotomy level; T1S: T1 slope; TPA: T1 pelvic angle; TK: thoracic kyphosis; LL: lumbar lordosis; C7 SVA: C7 sagittal vertical axis; PI: pelvic incidence; PT: pelvic tile; SS: sacral slope).

The following radiographic parameters were measured preoperatively (Pre), postoperatively (Post), and at 2 year follow‐up (Final): sagittal Cobb of T10 to L2 thoraco‐lumbar kyphosis (TLK); sagittal Cobb of segmental kyphosis (SK); mean value of anterior and middle body height for osteotomy level (AOH/MOH); T1 slope (T1S); T1 pelvic angle (TPA); thoracoc kyphosis (TK); lumbar lordosis (LL); C7 sagittal vertical axis (C7 SVA); pelvic incidence (PI); PT; and sacral slope (SS). Three experienced spinal surgeons, blinded to the study design, independently measured radiographic parameters using the PACS system (Centricity RIS/PACS, GE Healthcare), and the average of their measurements was used for the analysis. The detailed parameters are shown in Figure 2.

### 2.5 Clinical Assessment

The intraoperative and post‐operative complications were recorded for each patient. Patient‐reported outcome measurements (PROMs) were assessed using the Oswestry Disability Index (ODI) and Scoliosis Research Society‐22 (SRS‐22) questionnaire. Data from the questionnaires, independently completed by the patients preoperatively and at each follow‐up, were analyzed.

### 2.6 Statistical Analysis

Categorical variables were compared using the chi‐squared test. Continuous variables were tested for normality (Shapiro–Wilk test) and expressed as mean ± SD or median (IQR). Independent *t* tests or Mann–Whitney *U* tests were used for between‐group comparisons, and paired *t* tests or Wilcoxon signed‐rank tests were used for intragroup comparisons. The inter‐rater reliability of radiographic measurements was excellent (ICC = 0.950). Statistical significance was set at *p* < 0.05. Analyses were performed using SPSS 23, and graphs were created using GraphPad Prism 10.

## 3 Results

### 3.1 Demographics and Operative Parameters

Fifty‐four patients (42 females and 12 males) were included in the 3CO group, and 21 (17 females and 4 males) were included in the LCR‐A group. Comparison analysis showed significant differences between the two groups in terms of age (*p* = 0.001), fusion level (*p* = 0.001), operation time (*p* = 0.001), estimated blood loss (*p* = 0.001), and bone density score (*p* = 0.001). The general data are presented in Table 1.

**TABLE 1:** Demographic and operative parameters between two groups.

Demographic	3CO (*N* = 54)	LCR‐A (*N* = 21)	*p*
Gender(F/M) (*N*)	42:12	17:4	0.424
Age(year)	67.1 ± 3.2	77.8 ± 5	**0.001**
BMI (kg/m^2^)	22.3 ± 3.6	23.3 ± 2.4	0.330
Fusion level (*vertebra*)	8.4 ± 3	5.4 ± 0.7	**0.001**
Osteotomy level (*N*)			
T10	3	2	—
T11	6	4	—
T12	16	7	—
L1	25	6	—
L2	4	2	—
EBL (mL)	1226.5 ± 648	166.7 ± 100.8	**0.001**
OP time (min)	389.9 ± 101	263.8 ± 99.6	**0.001**
*T*‐score	−2.2 ± 0.9	−3.3 ± 0.4	**0.001**

*Note*: The value was listed as mean ± standard deviation. Boldface values indicate statistically significant.

Abbreviations: 3CO: three‐column osteotomy; EBL: estimated blood loss; LCR‐A: lateral column realignment with ALL release; OP: operation.

### 3.2 Radiographic Outcomes

At pre‐operation, the sagittal Cobb of TLK was 50.7° ± 13.7° in the 3CO group, 33.9° ± 6° in the LCR‐A group, and the sagittal Cobb of SK was 25° ± 9.1° in the 3CO group, 40.5° ± 3.8° in the LCR‐A group, with a significant difference observed between the two groups (*p* = 0.001). There were no statistical differences between the two groups in terms of other parameters (*p* > 0.050).

For patients in the 3CO group, the average postoperative values were 13° ± 11.4° for the sagittal Cobb angle of TLK, −2.4° ± 7° for the sagittal Cobb angle of SK, and 27.3 ± 6.3 mm for OH. For patients in the LCR‐A group, the average postoperative values were 11.1° ± 3.1° for the sagittal Cobb angle of TLK, 15.8° ± 2.2° for the sagittal Cobb angle of SK, and 32.1 ± 3.8 mm for OH. Significant differences were observed in the sagittal Cobb angle of SK (*p* = 0.001) and OH (*p* = 0.014).

At 2‐year last follow‐up, the sagittal Cobb of TLK increased to 16.2° ± 10.1° in the 3CO group, 12.4° ± 2.8° in the LCR‐A group, while the sagittal Cobb of SK increased to −0.9° ± 7° in the 3CO group, 16.9° ± 2.3° in the LCR‐A group. The OH decreased to 27.5 ± 6.0 mm in the 3CO group, 31.2 ± 3.6 mm in the LCR‐A group. Significant differences were observed in the sagittal Cobb angle of SK (*p* = 0.001) and OH (*p* = 0.044). The details of the radiographic parameters preoperatively, postoperatively, and 2‐year last follow‐up are shown in Table 2.

**TABLE 2:** Radiographic comparison between preoperative (*Pre*), postoperative (*Post*), and 2‐year follow‐up (*Final*).

		T1S (°)	TK (°)	LL (°)	PT (°)	SS (°)	TPA (°)	SVA (mm)	TLK Cobb (°)	SK Cobb (°)	OH (mm)
*Pre*	3CO	37.1 ± 14.1	46.3 ± 20.4	28.6 ± 28.5	32.3 ± 12.4	18.6 ± 11.3	33.2 ± 17.1	84 ± 69.8	50.7 ± 13.7	25 ± 9.1	25.7 ± 5.2
	LCR‐A	44.5 ± 6.9	49.7 ± 8.1	25.5 ± 12.5	29.8 ± 10	17.8 ± 10.5	33.4 ± 5.2	100.2 ± 30.5	33.9 ± 6	40.5 ± 3.8	23.8 ± 2
	*p*	0.080	0.570	0.699	0.521	0.413	0.959	0.437	**0.001**	**0.001**	0.220
*Post*	3CO	29.3 ± 11.1	38.1 ± 10.5	37.5 ± 15.7	24.3 ± 9.8	26.7 ± 10.7	22.4 ± 10.2	49.2 ± 52.1	13 ± 11.4	−2.4 ± 7	27.3 ± 6.3
	LCR‐A	34.6 ± 6.8	33.1 ± 5.2	47.5 ± 10.9	25 ± 7	33.1 ± 7.9	21.8 ± 2.9	68.3 ± 20.4	11.1 ± 3.1	15.8 ± 2.2	32.1 ± 3.8
	*p*	0.117	0.115	**0.041**	0.830	0.054	0.838	0.219	0.582	**0.001**	**0.014**
*Final*	3CO	35.7 ± 14.8	42.4 ± 12.7	33.4 ± 24.1	27.9 ± 12.2	24.9 ± 12	27.5 ± 15.8	73.3 ± 71.4	16.2 ± 10.1	−0.9 ± 7	27.5 ± 6
	LCR‐A	35.3 ± 7.4	35.3 ± 5.1	47.2 ± 9.5	26 ± 6.4	32.2 ± 7.8	25.3 ± 5	73.4 ± 19.3	12.4 ± 2.8	16.9 ± 2.3	31.2 ± 3.6
	*p*	0.932	0.063	0.057	0.594	0.052	0.640	0.997	0.203	**0.001**	**0.044**

*Note*: The value was listed as mean ± standard deviation. Boldface values indicate statistically significant.

Abbreviations: 3CO: three‐column osteotomy; LCR‐A: lateral column realignment with ALL release.

### 3.3 Pre to Postoperative and Final Reciprocal Changes Comparison of 3CO Versus LCR‐A

**FIGURE 3:**
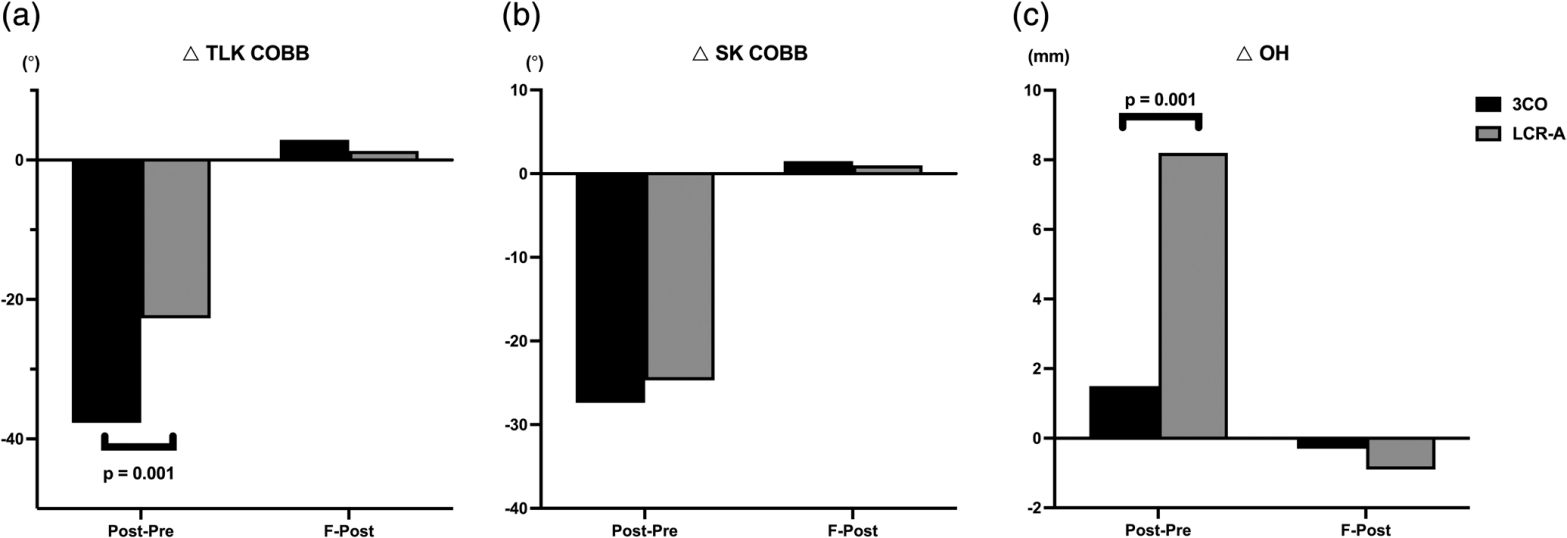
Reciprocal changes comparison between preoperative (*Pre*), postoperative (*Post*), and 2‐year follow‐up (*Final*).

Significant differences between the two groups in changes in parameters (pre to post) were found in the sagittal Cobb of TLK, OH (*p* = 0.001), and LL and SS (*p* = 0.046 and 0.037, respectively). However, no significant difference in the changes in parameters (post to final) was observed between the two groups. The details of reciprocal changes are presented in Table 3 and Figure 3.

**TABLE 3:** Reciprocal changes comparison between preoperative (*Pre*), postoperative (*Post*), and 2‐year follow‐up (*Final*).

		T1S (°)	TK (°)	LL (°)	PT (°)	SS (°)	TPA (°)	SVA (mm)	TLK Cobb (°)	SK Cobb (°)	OH (mm)
*Post—Pre*	3CO	−8.4 ± 11.2	−8.1 ± 16.4	8.9 ± 25.1	−7.9 ± 10.2	8.1 ± 10.3	−10.7 ± 13.5	−34.8 ± 55.4	−37.7 ± 14.2	−27.4 ± 10.2	1.5 ± 5.3
	LCR‐A	−10 ± 2.1	−16.6 ± 5.9	21 ± 3.9	−4.8 ± 5.5	16.4 ± 4.9	−11.6 ± 4	−31.9 ± 35.2	−22.7 ± 7.4	−24.7 ± 5.3	8.2 ± 3.3
	*p*	0.624	0.085	**0.046**	0.312	**0.037**	0.827	0.861	**0.001**	0.373	**0.001**
*Final–Post*	3CO	6 ± 14	4.3 ± 8.8	−4 ± 15.9	2.9 ± 7.5	−1.7 ± 7.9	4.7 ± 11.1	24.9 ± 56.6	2.9 ± 6.1	1.5 ± 4.3	−0.3 ± 4.5
	LCR‐A	0.7 ± 1.2	2.2 ± 0.5	−0.3 ± 3.3	1 ± 2.4	−1 ± 2.3	3.5 ± 3.8	5.1 ± 3.9	1.3 ± 0.8	1.1 ± 0.9	−0.9 ± 0.6
	*p*	0.196	0.412	0.423	0.385	0.770	0.714	0.234	0.347	0.735	0.660

*Note*: The value was listed as mean ± standard deviation. Boldface values indicate statistically significant.

Abbreviations: 3CO: three‐column osteotomy; LCR‐A: lateral column realignment with ALL release.

### 3.4 Clinical Outcomes

In the 3CO group, the average scores of ODI and SRS‐22 were 44.9 ± 19.2 and 2.6 ± 0.6, preoperatively, and then showed significant improvements to 36.2 ± 23.6 and 3.3 ± 0.8, respectively (*p* = 0.013 and 0.001). In the LCR‐A group, the average scores of ODI and SRS‐22 total were 71.5 ± 6.0 and 3.1 ± 0.2, preoperatively, and then showed significant improvements to 12.7 ± 3.3 and 4.0 ± 0.1, respectively (*p* = 0.001 and 0.001). Although both the 3CO and LCR‐A groups demonstrated statistically significant improvements in ODI and SRS‐22 scores—including all subdomains—at the final follow‐up compared to preoperative values, the LCR‐A group exhibited significantly better patient‐reported outcome measures (PROMs) at the final follow‐up and greater improvements in ODI compared to the 3CO group (*p* = 0.001). Detailed data are listed in Table 4.

**TABLE 4:** Comparison of ODI and SRS‐22 measures between preoperative (*Pre*) and 2‐year follow‐up (*Final*).

	3CO	LCR‐A	*p*
*Pre* ODI	44.9 ± 19.2	71.5 ± 6	**0.001**
*Final* ODI	36.2 ± 23.6	12.7 ± 3.3	**0.001**
*p* (*Final* vs. *Pre*)	**0.013**	**0.001**	
*Final—Pre* ODI	−9.2 ± 22.8	−58.8 ± 5.4	**0.001**
*Pre* SRS22 function	2.5 ± 0.7	3.3 ± 0.4	**0.001**
*Final* SRS22 function	3.1 ± 0.8	3.8 ± 0.1	**0.003**
*p* (*Final* vs. *Pre*)	**0.001**	**0.001**	
*Final—Pre* SRS22 function	0.6 ± 0.8	0.6 ± 0.4	0.812
*Pre* SRS22 pain	3.1 ± 0.8	3.3 ± 0.4	0.373
*Final* SRS22 pain	3.7 ± 1.1	3.9 ± 0.1	0.402
*p* (*Final* vs. *Pre*)	**0.001**	**0.001**	
*Final—Pre* SRS22 Pain	0.6 ± 1	0.7 ± 0.4	0.766
*Pre* SRS22 SI	2 ± 0.7	2.9 ± 0.2	**0.001**
*Final* SRS22 SI	3.2 ± 0.9	4.1 ± 0.1	**0.002**
*p* (*Final* vs. *Pre*)	**0.001**	**0.001**	
*Final—Pre* SRS22 SI	1.3 ± 1	1.2 ± 0.2	0.838
*Pre* SRS22 MH	2.5 ± 0.9	3.1 ± 0.3	**0.041**
*Final* SRS22 MH	3.2 ± 1	4.1 ± 0.1	**0.005**
*p* (*Final* vs. *Pre*)	**0.001**	**0.001**	
*Final—Pre* SRS22 MH	0.8 ± 1.2	1 ± 0.4	0.441
*Pre* SRS22 satisfaction	3.2 ± 0.9	3 ± 0.2	0.388
*Final* SRS22 satisfaction	3.4 ± 1	4.1 ± 0.1	**0.011**
*p* (*Final vs. Pre*)	0.418	**0.001**	
*Final—Pre* SRS22 Satisfaction	0.3 ± 1.2	1.2 ± 0.2	**0.015**
*Pre* SRS22 total	2.6 ± 0.6	3.1 ± 0.2	**0.003**
*Final* SRS22 total	3.3 ± 0.8	4 ± 0.1	**0.006**
*p* (*Final* vs. *Pre*)	**0.001**	**0.001**	
*Final—Pre* SRS22 total	0.8 ± 0.8	0.9 ± 0.2	0.448

*Note*: The value was listed as mean ± standard deviation. Boldface values indicate statistically significant.

Abbreviations: 3CO: three‐column osteotomy; LCR‐A: lateral column realignment with ALL release.

### 3.5 Propensity Matching With Age

Propensity score matching analysis also demonstrated that the LCR‐A group achieved superior correction in TPA (*p* = 0.045) and OH (*p* = 0.001), with a comparable degree of SK correction (*p* = 0.660). However, the 3CO group remained superior in terms of TLK correction (*p* = 0.001). Detailed data are presented in Table 5.

**TABLE 5:** The comparison based on age‐matching.

Group	Before propensity score matching			After propensity score matching		
	3CO (*N* = 54)	LCR‐A (*N* = 21)	*p*	3CO (*N* = 10)	LCR‐A (*N* = 10)	*p*
Gender (F/M) (*N*)	42:12	17:4	0.424	8:2	6:4	0.628
Age(year)	67.1 ± 3.2	77.8 ± 5	**0.001**	75.2 ± 3.3	76 ± 2.9	0.576
BMI (kg/m^2^)	22.3 ± 3.6	23.3 ± 2.4	0.330	21.3 ± 1.5	23.3 ± 2.5	0.065
Fusion level (*vertebra*)	8.4 ± 3	5.4 ± 0.7	**0.001**	8.4 ± 3.4	5.5 ± 0.7	**0.018**
EBL (mL)	1226.5 ± 648	166.7 ± 100.8	**0.001**	1175 ± 808.9	175 ± 103.4	**0.001**
OP time (min)	389.9 ± 101	263.8 ± 99.6	**0.001**	367.4 ± 99.7	260.5 ± 108.5	**0.034**
*T*‐score	−2.2 ± 0.9	−3.3 ± 0.4	**0.001**	−2.1 ± 0.6	−3.3 ± 0.4	**0.002**
*Post—Pre*						
TPA (°)	−10.7 ± 13.5	−11.6 ± 4	0.827	−5.1 ± 9	−11.9 ± 4.3	**0.045**
TLK Cobb (°)	−37.7 ± 14.2	−22.7 ± 7.4	**0.001**	−38.2 ± 10.2	−23 ± 7.8	**0.001**
SK Cobb (°)	−27.4 ± 10.2	−24.7 ± 5.3	0.373	−26 ± 9.8	−24.4 ± 5.8	0.660
OH (mm)	1.5 ± 5.3	8.2 ± 3.3	**0.001**	0.8 ± 3.3	8.3 ± 2.9	**0.001**

*Note*: The value was listed as mean ± standard deviation. Boldface values indicate statistically significant.

Abbreviations: 3CO: three‐column osteotomy; LCR‐A: lateral column realignment with ALL release.

### 3.6 Illustrated Case

**FIGURE 4:**
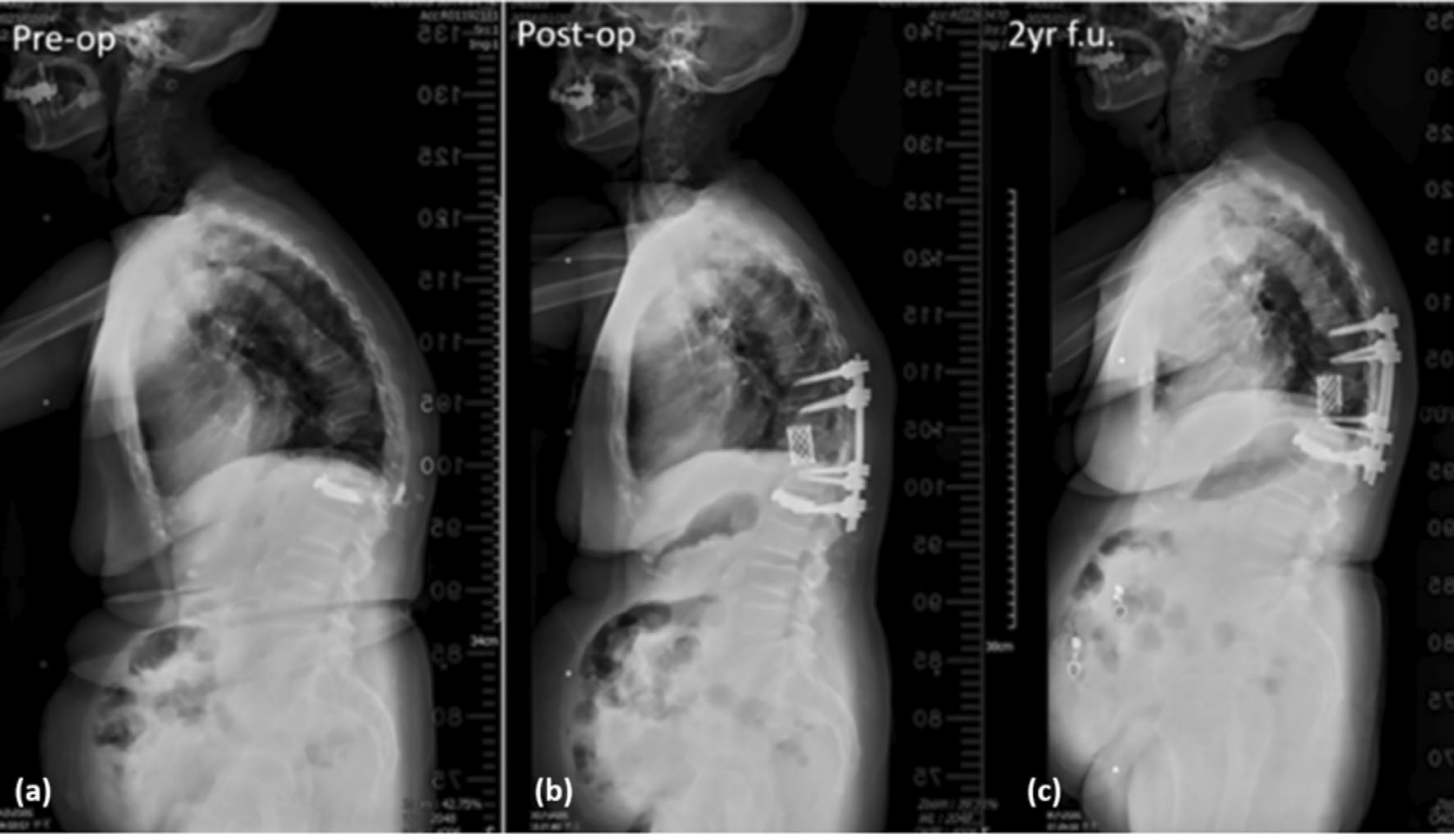
(a) An 81‐year‐old female presented with thoracolumbar kyphosis secondary to OVF and decompensated sagittal imbalance (TLK: 39°; SK: 37.7°; OH: 26.8 mm). (b) She underwent T11 LCR‐A combined with PCO and PSF (T9–L1) under navigation in the lateral decubitus position. The procedure achieved significant sagittal correction (TLK: 22.8°; SK: 4°; OH: 33.7 mm), with an estimated blood loss of 200 mL and operative duration of 316 min. No drainage was required, and the patient was able to ambulate on postoperative day one. (c) Radiographic follow‐up at 2 years demonstrated well‐maintained correction (TLK: 24.5°; SK: 5°; OH: 32.5 mm).

An 81‐year‐old female with thoracolumbar kyphosis secondary to OVF underwent T11 LCR‐A plus PCO and PSF (T9–L1) with navigation in a single decubitus position. Preoperative TLK was 39°, SK 37.7°, and OH 26.8 mm; postoperative values improved to 22.8°, 4°, and 33.7 mm. Surgery (316 min, 200 mL blood loss) was completed without drainage. The patient ambulated on postoperative day 1, maintaining correction at 2 years. This case is illustrated in Figure 4.

### 3.7 Complications

A total of 28 complications occurred in 19 patients in the 3CO group, and 5 complications occurred in 4 patients in the LCR‐A group (*p* = 0.048). New‐onset neurological deficits were reported in five 3CO patients, whereas two LCR‐A patients had pre‐existing deficits without postoperative deterioration. Among the five patients who developed new‐onset neurological deficits following 3CO, all were classified as Frankel C. After 2 years of rehabilitation, three of these patients improved to Frankel D. In the LCR‐A group, two patients had pre‐existing neurological deficits, both classified as Frankel C. Following surgery and 2 years of rehabilitation, one patient improved to Frankel D, and the other to Frankel E. Certain complications, including surgical site infections (SSI), hematoma, DVT, ischemic heart disease, and ileus, were exclusively observed in the 3CO group. Delirium is more frequent in the 3CO group than in the LCR‐A group (*p* = 0.021). Regarding postoperative complications in the 3CO group, three cases of SSI were managed with serial debridement, vacuum‐assisted closure therapy, and targeted antibiotic treatment until complete wound healing. One case of postoperative hematoma was treated with surgical evacuation and placement of multiple negative pressure drainage tubes, leading to full recovery. One case of DVT was treated with an anti‐coagulation agent. Nine cases of postoperative delirium were managed with psychiatric consultation and pharmacologic therapy; five patients recovered before discharge, while the remaining four recovered during outpatient follow‐up. One patient developed ischemic heart disease postoperatively and underwent emergency percutaneous coronary intervention with anticoagulation therapy, followed by intensive care monitoring until normalization of troponin‐I levels. Four cases of postoperative ileus were managed with nasogastric decompression, bowel rest, and pharmacologic bowel evacuation, resulting in recovery after several days. Two cases of pneumonia and three cases of UTI were treated successfully with antibiotics prior to discharge. Detailed data are presented in Table 6.

**TABLE 6:** The summary of post‐operative complications in two groups.

Complication (*N*/%)	3CO (*N* = 54)	LCR‐A (*N* = 21)	*p*
Neurological Deficient	5 (9.3)	2 (9.5)	0.981
SSI	3 (5.6)	0	—
Hematoma	1 (1.9)	0	—
Delirium	8 (14.8)	1 (4.8)	**0.021**
DVT	1 (1.9)	0	—
Cardiac	1 (1.9)	0	—
Pneumonia	2 (3.7)	1 (4.8)	0.458
Gastro‐Ileus	4 (7.4)	0	—
UTI	3 (5.6)	1 (4.8)	0.561
Total	19 (35.2)	4 (19.0)	**0.048**

*Note*: Boldface values indicate statistically significant.

Abbreviations: 3CO: three‐column osteotomy; DVT: deep vein thrombosis; LCR‐A: lateral column realignment with ALL release; SSI: surgical site infection; UTI: urinary tract infection.

## 4 Discussion

### 4.1 Estimated Blood Loss and Fusion Segments

In the present study, patients in the LCR‐A group tended to be older than those in the 3CO group (77.8 ± 5 vs. 67.1 ± 3.2, *p* = 0.001); however, they exhibited significantly reduced EBL (166.7 ± 100.8 vs. 1226.5 ± 648, *p* = 0.001) and shorter OPT (263.8 ± 99.6 vs. 389.9 ± 101, *p* = 0.001). These factors may contribute to the lower incidence of postoperative complications. A study by Licina et al. [25] highlighted that patients undergoing moderate to major spinal surgery are at risk of substantial blood loss, often necessitating fluid resuscitation and blood product transfusion. Willner et al. [26] emphasized that any therapeutic modality capable of reducing blood loss and the need for blood transfusion would be highly beneficial for spinal surgery patients. Furthermore, the LCR‐A group required fewer fused spinal segments than the 3CO group (5.4 ± 0.7 vs. 8.4 ± 3, *p* = 0.001), which may also play a role in reducing surgical morbidity. The study by Jonathan A. Ledesma et al. [27] demonstrated that short‐construct fusions may yield satisfactory improvements in patient‐reported outcome measures (PROMs), particularly in the ODI and the Visual Analog Scale (VAS) leg pain score. Moreover, in appropriately selected patients, this approach may reduce the length of hospital stay, revision surgery rates, and likelihood of non‐home discharge. Lai et al. [28] demonstrated that short‐segment fixation combined with kyphoplasty can achieve circumferential stabilization for osteoporotic thoracolumbar burst fractures with reduced surgical invasiveness and fewer involved motion segments. Based on the aforementioned studies, the LCR‐A technique may be a more advantageous surgical option for elderly patients.

### 4.2 Muscle Damage and Bony Resection

Compared with 3CO, which necessitates extensive posterior muscle dissection and wide bony resection, including the lamina, pedicle, and partial vertebral body, the osteotomy plane and incomplete muscle stripping in 3CO are major sources of uncontrollable intraoperative bleeding. In contrast, the LCR‐A approach involves only blunt dissection through the retroperitoneal or retropleural fascia, a technique that is associated with minimal bleeding owing to its muscle‐sparing nature. Moreover, extensive muscle dissection is a major source of postoperative pain [29, 30], which likely contributes to the significantly higher postoperative ODI observed in the 3CO group than in the LCR‐A group (36.2 ± 23.6 vs. 12.7 ± 3.3). The study by Kim [29] highlighted that spine surgery inherently results in damage to the surrounding musculature, characterized by muscle atrophy and subsequent functional impairment. This damage is primarily due to direct injury from the dissection and detachment of the tendinous insertions from the posterior spinal elements. Additionally, denervation serves as a further mechanism contributing to postoperative muscle degeneration and atrophy. Dickerman et al. [30] indicated that minimally invasive anterior lumbar interbody fusion (ALIF) with percutaneous pedicle screw fixation results in less muscle damage than posterior lumbar interbody fusion (PLIF). Supporting this, biochemical evidence showed a 5.1‐fold increase in creatine phosphokinase (CPK) levels in the ALIF group, whereas the PLIF group exhibited an 8.7‐fold increase.

Moreover, the LCR‐A procedure takes advantage of ALL release to enhance disc flexibility, enabling partial correction of the deformity without the need for extensive bone cutting. This reduction in the osteotomy surface area further contributes to the significant decrease in blood loss observed in the LCR‐A group compared to that in the 3CO group.

### 4.3 Potential Risks Associated With Patient Positioning

3CO with long‐segment fusion requires patients to undergo surgery in the prone position, which imposes considerable cardiopulmonary stress and increases the risk of spinal cord perturbation during repositioning. In a study conducted by Willner et al. [26], it was noted that the prone position was associated with increased intra‐abdominal pressure and, consequently, a higher intraoperative blood loss than the lateral jackknife position. In a study by Lee et al. [31], it was found that a higher baseline Pleth Variability Index was associated with the development of hypotension prior to surgical incision in the prone position.

Based on the study data, although there was no statistically significant difference in neurological deficits between the 3CO and LCR‐A groups, it is noteworthy that the two cases of neurological deficits in the LCR‐A group were pre‐existing before surgery. These patients were selected for the lateral approach specifically because of their preoperative neurological impairments. Excluding these two cases, no new‐onset neurological deficits were observed postoperatively in the LCR‐A group. This finding suggests that the lateral decubitus approach employed in LCR‐A may offer a protective advantage against positional spinal cord insult, particularly when compared with the prone positioning required in 3CO procedures. By contrast, the LCR‐A procedure can be performed in the lateral decubitus position, thereby minimizing these risks and enhancing the safety and feasibility of surgical correction in the elderly population.

Although the preoperative TK in the PSO and LCR‐A groups was 46.3° ± 20.42° and 49.7° ± 8.1°, respectively, it is important to highlight that the sagittal Cobb angle of TLK was 50.7° ± 13.7° in the 3CO group and 33.9° ± 6° in the LCR‐A group, while the sagittal Cobb angle of SK was 25° ± 9.1° in the 3CO group and 40.5° ± 3.8° in the LCR‐A group. These data indicate that the patient population in this study primarily exhibited relatively localized sharp kyphotic deformities at the thoracolumbar junction. As shown in Table 2, the preoperative SK was significantly greater in the LCR‐A group than in the 3CO group (*p* = 0.001), while the TLK Cobb angle was significantly smaller (*p* = 0.001). These findings indicate that the kyphotic deformity in the LCR‐A group presented with a more acute angular deformity compared to the 3CO group. This morphological characteristic partly explains the surgical rationale for employing the lateral approach in the LCR‐A group, as it may reduce the risk of irreversible spinal cord injury caused by apex compression due to gravity during prone positioning under general anesthesia [19, 32]. A study conducted by Yoshida et al. [32] identified that a transcranial motor‐evoked potential (Tc‐MEP) alert during patient repositioning serves as a critical indicator of potential spinal cord injury resulting from alignment changes in the upper thoracic spine. Jin et al. [19] indicated that in patients with kyphotic deformity, those with type C spinal cord morphology, especially the C+ subtype, are at a significantly increased risk of intraoperative neurophysiological monitoring (IONM) events during kyphosis correction in the prone position.

### 4.4 Risk of Neurological Injury Associated With Osteotomy Techniques

Furthermore, in contrast to the 3CO group, which primarily relied on posterior column shortening techniques for correction, the LCR‐A group achieved deformity correction by restoring the original vertebral body height lost due to old compression fractures (Postoperative OH: 32.1 ± 3.8 vs. 27.3 ± 6.3, *p* = 0.014). This approach is more aligned with restoring the native shape of the spinal canal and may reduce the risk of dural buckling associated with excessive posterior shortening. The study by Turner et al. [33] highlighted that in patients with severe kyphosis, the elongation of the spinal cord also results in stretching of the associated blood vessels, potentially leading to a reduction in vessel caliber and compromised blood flow. While the initial phase of 3CO gap closure appeared to reposition the vessels more favorably, thereby enhancing perfusion, subsequent stages of reduction may have caused vessel “kinking,” contributing to the observed decrease in spinal cord perfusion. Additionally, the surgical technique of LCR‐A, compared to that of 3CO, avoids direct manipulation of the spinal cord with surgical instruments, thereby reducing the risk of iatrogenic dural injury during the procedure. Despite employing a different corrective strategy, the amount of correction achieved in SK Cobb was comparable between the two groups (Post‐Pre SK Cobb: −24.7 ± 5.3 vs. −27.4 ± 10.2, *p* = 0.373). Detailed data are shown in Figure 3.

### 4.5 Propensity Matching With Age

Before propensity score matching, the LCR‐A group had significantly higher age. Although the matching was performed based on age—recognized risk factors for prolonged operative time and increased blood loss—the LCR‐A group still demonstrated significantly shorter operative times and lower estimated blood loss. These findings suggest that the LCR‐A surgical approach may represent a faster and less invasive option for the treatment of thoracolumbar kyphosis in septuagenarians.

### 4.6 Strengths and Limitations

Strengths of the study include: First, it provides a direct and detailed comparison between the traditional 3CO and the newer, minimally invasive LCR‐A, specifically in elderly patients with thoracolumbar kyphosis secondary to OVF. This fills an important gap in the current literature. Second, the focus on septuagenarian patients underscores the clinical relevance and real‐world applicability of the findings, offering an evidence‐based surgical alternative tailored to a particularly vulnerable demographic. Third, the use of propensity score matching for age enhances the validity of the comparative analysis by reducing bias, particularly given the higher mean age in the LCR‐A group.

Limitations of the study include the relatively small sample size in the LCR‐A group, the relatively short 2‐year follow‐up period, and heterogeneity in curve patterns and fusion strategies, which may limit the robustness of the statistical analysis. Future multicenter studies with larger cohorts and longer follow‐up durations are warranted to provide a more comprehensive evaluation of the efficacy and safety of LCR‐A in the management of rigid thoracolumbar kyphosis.

## 5 Conclusions

The single‐position navigated LCR‐A technique presents a valuable alternative to the traditional 3CO technique for managing thoracolumbar rigid kyphosis secondary to OVF. Moreover, the LCR‐A approach has demonstrated reduced estimated blood loss and a shorter operative duration. Additionally, this method is associated with fewer postoperative complications, effective pain relief, and accelerated recovery even in elderly patients with complex medical comorbidities.

## Author Contributions

**Xue‐Peng Wei:** provided administrative and intellectual support. **Hung‐Lun Hsieh:** acquisition of data; analysis and interpretation of data; **Qing‐De Wang:** acquisition of data; analysis and interpretation of data; **Yi‐Hsun Huang:** acquisition of data; analysis and interpretation of data; **Erh‐Ti Ernest Lin and Chen‐Wei Yeh:** acquisition of data; analysis and interpretation of data. All authors read and approved the final manuscript; **Yuan‐Shun Lo:** the conception and design of the study; analysis and interpretation of data; drafting the article; revising it critically for important intellectual content; final approval of the version to be submitted; final approval of the version to be submitted.

## Ethics Statement

Consent was obtained from all study participants, and approval was obtained from the Institutional Review Board of China Medical University Hospital (CMUH111‐REC1‐128).

## Conflicts of Interest

The authors declare no conflicts of interest.

## Data Availability

The data that support the findings of this study are available on request from the corresponding author. The data are not publicly available due to privacy or ethical restrictions.
